# The Sound of Success: Investigating Cognitive and Behavioral Effects of Motivational Music in Sports

**DOI:** 10.3389/fpsyg.2017.02026

**Published:** 2017-11-21

**Authors:** Paul Elvers, Jochen Steffens

**Affiliations:** ^1^Max Planck Institute for Empirical Aesthetics, Frankfurt am Main, Germany; ^2^Audio Communication Group, Technische Universität Berlin, Berlin, Germany

**Keywords:** music, motivation, self-enhancement, self-esteem, sports, risk taking, motor coordination

## Abstract

Listening to music before, during, or after sports is a common phenomenon, yet its functions and effects on performance, cognition, and behavior remain to be investigated. In this study we present a novel approach to the role of music in sports and exercise that focuses on the notion of musical self-enhancement (Elvers, 2016). We derived the following hypotheses from this framework: listening to motivational music will (i) enhance self-evaluative cognition, (ii) improve performance in a ball game, and (iii) evoke greater risk-taking behavior. To evaluate the hypotheses, we conducted a between-groups experiment (*N* = 150) testing the effectiveness of both an experimenter playlist and a participant-selected playlist in comparison to a no-music control condition. All participants performed a ball-throwing task developed by Decharms and Davé (1965), consisting of two parts: First, participants threw the ball from fixed distances into a funnel basket. During this task, performance was measured. In the second part, the participants themselves chose distances from the basket, which allowed their risk-taking behavior to be assessed. The results indicate that listening to motivational music led to greater risk taking but did not improve ball-throwing performance. This effect was more pronounced in male participants and among those who listened to their own playlists. Furthermore, self-selected music enhanced state self-esteem in participants who were performing well but not in those who were performing poorly. We also discuss further implications for the notion of musical self-enhancement.

## Introduction

Many people across the world, from occasional runners and gym goers to world-class athletes, integrate music into their competitions and workout routines. While people's reasons and motivations for listening to music before, after, or during sports are manifold and diverse (Laukka and Quick, 2011), one salient function of music is to help athletes gain self-confidence and motivation (Karageorghis and Priest, 2012a,b), of which numerous examples exist (see e.g., Terry and Karageorghis, 2011). For example, during the Olympic games in Beijing 2008, the American swimmer Michael Phelps listened to music on his portable music device until 2 min before his competition to get himself into the right mindset (Terry and Karageorghis, 2011). Another example is the Maori battle cry “Haka,” which is regularly performed by the New Zealand national rugby team prior to competitions (World-Rugby, 2015). It serves as a display of power and confidence and also allows the players to get into the right mindset prior to competitions. Although a concise theory of the motivational function of music in sports is still lacking, some conceptual efforts have been made: Karageorghis and Priest (2012a) argue that the motivational function of music relies on both intrinsic musical factors such as rhythm, melody, and harmony, and external factors such as cultural impact and extra-musical associations. However, the mechanisms that explain how music exerts a motivational function are as yet poorly understood (Karageorghis and Priest, 2012a).

We suggest that the psychological processes that are linked to motivation and emotion play an important role for understanding the functions and effects of music in sports and exercise. We follow up on a novel conception of motivational music connecting it to self-enhancement processes: It has been suggested that the empowering function of listening to music may be an esthetic surrogate for social interaction that allows self-regulative processes to occur (Elvers, 2016). While previous research has documented the use of music for mood and emotion regulation (Saarikallio, 2010; Krueger, 2014), Elvers (2016) suggests that self-evaluative attitudes and cognitive processes are also influenced by music. More specifically, this account suggests that listening to music fulfills self-enhancement needs, providing listeners with enhanced self-esteem, optimism, and self-worth. Initial experimental evidence suggests that listening to powerful music activates power-related cognition and behavior (Hsu et al., 2014) and that listening to empowering music enhances momentary self-esteem (Elvers et al., 2017). Following from this initial evidence, we assume that motivational music also exerts a positive influence on self-evaluative cognition and subsequent behavior.

### Musical self-enhancement

While the role of music in evoking emotional responses (Scherer and Zentner, 2001, 2008; Juslin and Västfjäll, 2008; Zentner et al., 2008) and its use for mood regulation (Saarikallio, 2008, 2010; Skanland, 2013; Carlson et al., 2015) have been a subject of considerable scientific interest, the question of how listening to music relates to changes in self-evaluative cognitions has rarely been discussed. This is surprising, given that self-evaluative cognitions and attitudes such as self-esteem (Rosenberg, 1965), self-confidence (Woodman and Hardy, 2003), and self-efficacy (Bandura, 1977) are considered to be sensitive to external stimuli, such as music. One exception can be found in Brown and Mankowski (1993), who showed that music-induced changes in mood affect self-evaluative cognitions. The notion of musical self-enhancement employs the idea that various external resources can be used to conduct self-enhancement processes (Sedikides and Gregg, 2008) and that music is one means of doing so (Elvers, 2016). Self-enhancement generally refers to the motive to see oneself positively as well as the process of gaining self-worth and self-esteem (Swann et al., 1989; Sedikides and Gregg, 2008). Within the framework of musical self-enhancement, we hypothesize that music that expresses a positive self-evaluative attitude will be most effective for self-enhancement. However, the self-enhancement effects are moderated by the listener's response to the music in terms of liking, familiarity, and empathy. While empirical evidence has been gathered with regard to changes in momentary (explicit) self-esteem (Elvers et al., 2017), it remains to be investigated how behavioral aspects relating to the notion of self-enhancement are influenced by listening to motivational music.

### Music and performance enhancement

Regarding the improvement of performance through music listening, empirical evidence corroborates the ergogenic effects of listening to music in a variety of activity domains (for a review see Karageorghis and Priest, 2012a,b). It has been found that synchronous music improves performance in strenuous motor tasks such as treadmill walking (Karageorghis et al., 2009) and 400 m treadmill running (Terry et al., 2012). When physical exercise is combined with the intentional modulation of sound, this may further reduce the perceived effort of performing the task (Fritz et al., 2013). However, in the majority of studies that have investigated the role of music in simple endurance tasks, either the number of repetitions or the time needed for completing the task have served as the dependent measure (Karageorghis and Priest, 2012a,b). The role of music in complex sport activities that involve not only endurance but also motor coordination and accuracy (e.g., tennis, football, basketball) remains to be investigated. There is a need for study designs that test the effectiveness of music during the performance of complex tasks involving some kind of motor coordination.

Only a limited number of studies provide evidence in favor of an ergogenic effect of music on the performance of complex tasks involving motor coordination. One qualitative study on the use of music by tennis players suggests that music is frequently used for pre-performance enhancement in ball sports (Bishop et al., 2007). Other evidence comes from research on motor rehabilitation interventions for patients who suffer from Parkinson disease: it was found that music interventions can improve motor coordination and the accuracy of movements, not only in Parkinson patients but also in healthy control participants (Bernatzky et al., 2004). Further, two studies with small sample sizes (*N* = 3 and 15) provide evidence that music interventions can improve performance in netball shooting (Pates et al., 2003) and ball kicking (Silliman and French, 1993). How listening to music improves motor coordination by music has proved difficult to explain. One explanation stems from the fact that there is a strong connectivity between the auditory cortex and motor cortex areas, so that music might stimulate the brain areas responsible for motor coordination (Bernatzky et al., 2004). Rhythmic entrainment has also been proposed as a mechanism to explain how music improves motor coordination (Thaut et al., 2014). Performance enhancement in ball-throwing or ball-kicking tasks might also be explained by the induction of flow states (Pates et al., 2003) or improved self-confidence (Woodman and Hardy, 2003).

### Music and risk behavior

Another behavioral aspect that has received considerably less attention but is linked to both sport performance and self-evaluative cognition is risk behavior. Certain aspects of self-enhancement, such as a greater sense of power, may lead to riskier behavior (Anderson and Galinsky, 2006) and overconfident decision making (Fast et al., 2012). It has also been observed that on the trait level, self-esteem is associated with higher engagement in risky behaviors (Wild et al., 2004). As prospect theory (Kahneman and Tversky, 1979) suggests, people's risk preferences differ when mathematically identical options are framed positively as gains (increased risk preference) or negatively as losses (decreased risk preference). Prospect theory posits that framing effects account for the differences in risk preferences under positive and negative scenarios. It has been found that when given a choice between framing decisions involving risk positively or negatively, individuals with high self-esteem are more likely to impose a positive frame (McElroy et al., 2007). Thus, motivational music may lead to enhanced self-esteem, which in turn allows framing decisions regarding risks more positively, as gains rather than as losses. While a number of studies have investigated the effect that listening to music has on risk behavior, none of these have tested risk taking in a sports setting. Dey et al. (2006) and Brodsky (2002) showed that listening to music led to riskier behavior during a car-driving simulation. Halko and Kaustia (2015) showed, based on a gambling paradigm, that participants made riskier choices when listening to music they personally liked compared to music they personally disliked. Neuroscientific evidence suggests that personally liked music decreases loss aversion via differences in the value encoding of decisions under risk that correspond to enhanced activity in the amygdala and the striatum (Halko et al., 2015). When investigating risk behavior, gender differences also need to be taken into account. Across different domains and age groups, men are more likely than women to engage in risky behavior (Byrnes et al., 1999). These gender differences are even more pronounced when participants perform tasks involving physical skills such as playing shuffleboard or tossing rings onto pegs (Byrnes et al., 1999). Men are particularly more likely to take the opportunity to compete when given a choice between competing and not competing (Niederle and Vesterlund, 2007).

### The present study

In the present study, we seek to contribute to the notion of listening to music as self-enhancement by investigating the behavioral consequences of listening to motivational music. The aim was to test three main hypotheses, namely, that listening to motivational music (i) has a positive influence on performance in a ball game, (ii) enhances self-evaluative cognitions, and (iii) leads to riskier behavior. A between-groups experiment was designed to test the effectiveness of two different treatments of motivational music—experimenter-selected vs. self-selected—compared to a control group. For one condition, the selection of motivational music was guided by current scientific standards and musicological expertise, while the other condition had the individual participants select their own music, which would ensure greater ecological validity. In accordance with previous research on the psychological effects of listening to music (Chanda and Levitin, 2013), we further hypothesized that the effect would be more pronounced in the self-selected music condition. Since previous research suggests that there are gender differences in choices regarding risks, we also hypothesized that the influence of motivational music on risk behavior would be more pronounced in men. As a behavioral measure, a simple ball-throwing task was adopted from Decharms and Davé (1965). This paradigm was especially suitable since it allowed assessing both motor task performance and risk behavior. The task required throwing a volleyball from seven different distances into a funnel basket. While the distances were fixed in the first phase of the experiment, the participants themselves could choose the distances in the second phase.

## Methods

### Ethics statement

All experimental procedures were ethically approved by the Ethics Council of the Max Planck Society and were undertaken with the written informed consent of each participant.

### Recruitment and participants

The study was advertised through flyers and handouts at the Goethe University campus in Frankfurt am Main and in fitness gyms around the city. The flyers indicated that participants would receive 10 € per hour for their participation in the study. The target age span for the recruitment was between 18 and 35 years. Based on a pilot study (*N* = 10), the sample size was targeted to 150 by means of a power analysis using G^*^Power 3 (Faul et al., 2007). The final sample consisted of 150 participants (69.3% female; *M*_age_ = 23.40, range = 18–33). The majority (*N* = 136) of the participants were students (90.7%). Thirty-three (22%) of the participants indicated that they engaged in sports that involved handling a ball (e.g., volleyball, basketball, or handball) on a regular basis.

### Selection of motivational music

We decided to employ two different experimental conditions with motivational music, one in which the musical pieces were selected by the experimenters based on the expertise of musicologists and in line with current scientific approaches for selecting motivational music, and another in which the participants themselves selected the motivational music. While the former allowed more methodological rigor in terms of selecting musical pieces that would appropriately fit the intended function, the latter allowed more ecological validity and would ensure that the motivational music was appreciated by the listeners. For compilation of the self-selected playlist, a short online survey in which the participants compiled playlists containing 12 pieces of self-selected motivational music was administered to each participant prior to the study. The description of the survey task can be found in the Supplementary Material. An additional publication that considers auditory and semantic features of self-selected motivational music in detail is currently in preparation. For the experimenter-selected playlist, we had five music experts^1^ compile playlists of motivational music. Each expert was asked to consider aspects of motivational music stemming from previous approaches, such as the Brunel Music Rating Inventory (BMRI; Karageorghis et al., 1999) and the Brunel Music Rating Inventory-2 (BMRI-2; Karageorghis et al., 2006). The BMRI-2 guidelines for the selection of motivational music suggest that the following criteria are relevant: familiarity, fast tempo, pronounced rhythm and melody, associations with sports, or physical activity, and lyrics communicating self-competence, determination, and strength. Since the selection of motivational music and responses to it are highly idiosyncratic, we aimed to maximize participants' familiarity with and liking of the music by selecting songs that would match the cultural background of the population from which the participants were recruited. In subsequent group discussions, the five individual playlists were condensed to one playlist of motivational music containing 11 musical pieces (Table 1). The order of musical pieces was controlled by compiling five randomized playlists that were assigned to participants in a balanced order.

**Table 1:** List of musical pieces used for the experimenter playlist.

**Artist**	**Title**	**Album**	**Year**	**Duration**
Coldplay	Viva La Vida	Viva La Vida	2008	4:02
Ke$ha	TiK ToK	Animal + Cannibal	2010	3:19
Katy Perry	Roar	Prism	2013	3:42
Survivor	Eye of the Tiger	Rocky IV	1982	4:03
Limp Bizkit	Take A Look Around	Chocolate Starfish and the Hot Dog Flavored Water	2000	5:19
Eminem	Lose Yourself, Soundtrack Version	Curtain Call	2005	5:26
Queen	We Will Rock You, Remastered 2011	News Of The World	1977	2:02
Kanye West	Stronger	Graduation	2007	5:12
American Authors	Best Day of My Life	Best Day of My Life	2014	3:14
Capital Cities	Safe and Sound	Safe and Sound	2013	3:13
David Guetta	Titanium (feat. Sia)	Nothing But the Beat Ultimate	2012	4:05

### Procedure

**Figure 1:**
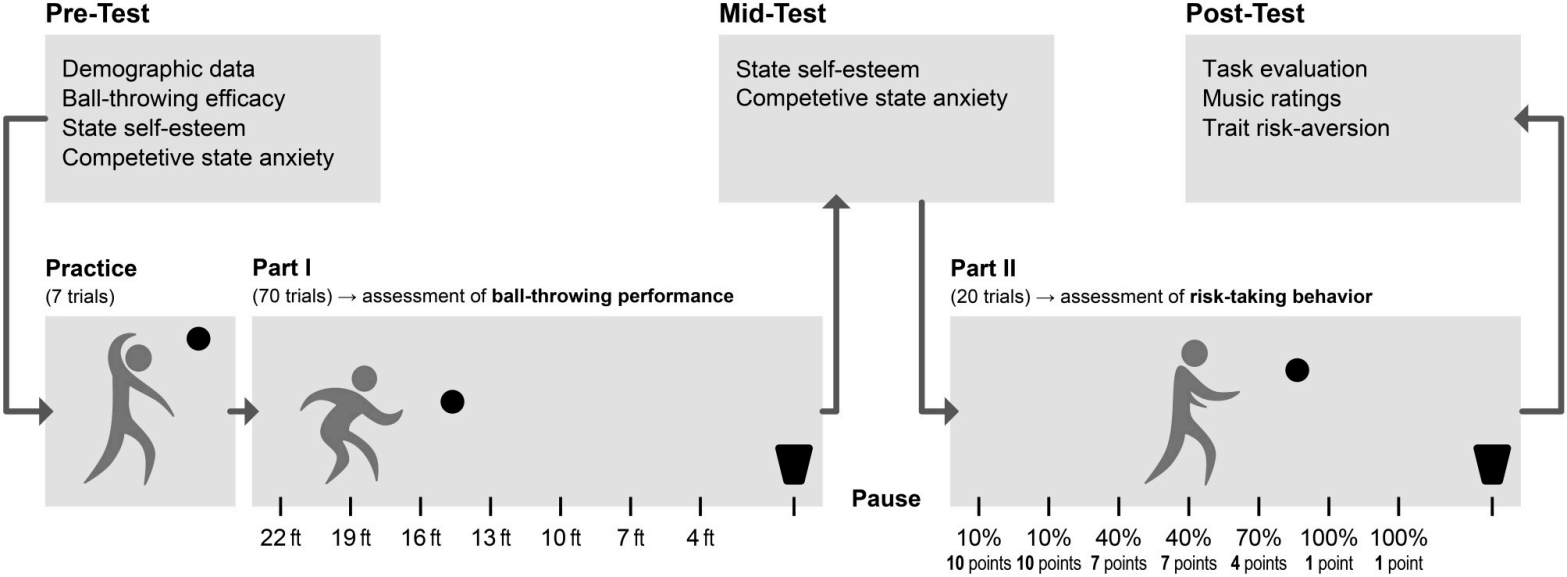
Illustration of the experimental procedure.

The data were collected from January to September 2016 in the laboratory rooms of the Max Planck Institute for Empirical Aesthetics in Frankfurt am Main, Germany. Participants were randomly assigned to one of the three experimental conditions, i.e., (i) experimenter-selected playlist, (ii) participant-selected playlist, or (iii) no-music control. Participants were invited to a room where the ball-throwing task was set up. For participants who were assigned to one of the music conditions, the playlist was already playing when they entered the room and was kept playing throughout the entire procedure until they were debriefed. The music was played at a medium level of loudness [≈70 dB(A)] that was kept constant across participants and throughout the procedure. The stereo loudspeaker system consisted of two Fohhn LX-150 speakers and two Fohhn X-22 active subwoofer. The experiment consisted of two main phases of the ball-throwing task, with overall performance being assessed during the first phase and risk taking during the second. Three questionnaires were also administered before, during, and after the ball throwing task. For an overview of the entire experimental procedure, see Figure 1.

### Measures

The behavioral ball-throwing paradigm allowed assessing two dependent variables: ball-throwing performance and risk taking. Both variables were assessed and calculated as documented in Decharms and Davé (1965). The experiment consisted of two main phases. In each phase, participants were asked to throw a volleyball into a funnel basket from seven different distances (4, 7, 10, 13, 16, 19, and 22 feet). For the assessment of ball-throwing performance, participants were asked to throw the ball 10 times from each distance, in the following order: 4, 10, 16, 22, 7, 13, and 19 feet. Since the number of trials, the distances, and the order were the same for all participants, their overall performance across the 70 trials could be compared between the experimental conditions. Before the beginning of the second phase, the experimenter calculated the average hitting ratio for each participant for each distance based on the participant's performance in the first phase. This weighing method allowed individual differences in ball-throwing accuracy to be accounted for. The hitting ratio was calculated for each of the seven distances as the number of successful trials divided by the overall number of trials. During the second phase, which aimed at assessing risk taking, participants had 20 trials (single ball tosses) for which they themselves chose the distances. After each ball toss, participants could choose the same, or another distance until all 20 trials were completed. For each successful trial, participants received incentivized points (0.05 € cents per point) corresponding to their previously determined hitting ratio. A hitting ratio of 100% would yield one point, 90% two points, and so on, up to 10 points for a 10% or 0% hitting ratio. The more difficult it was for participants to hit the basket from a given distance during the first phase, the more points they would receive for hitting it from that distance during the second phase. Risk taking was defined based on the participant's average hitting ratio during the first phase for the distance chosen for each of the twenty trials in the second phase. Additionally, we also assessed each participant's performance during the second phase as the sum of incentivized points the participant received for each successful trial in this phase. This would allow examining the relationship between choices regarding risk and overall gains in points.

In addition to the behavioral task, we obtained several subjective measures. Since the participants' training history appeared to be an important variable that could influence the effect of the motivational music (Brownley et al., 1995), the participants were asked whether they had previous experience in ball games (years of experience, experience level) and how they perceived their ball-throwing efficacy (α = 0.81). We also assessed trait risk aversion by using a domain-specific measure for risk in “sports and leisure,” the Risk Aversion–SOEP (RA-S; Dohmen et al., 2011). Two measures were administered to assess changes in self-evaluative cognitions. Both of these were administered as pre- and post-test measures before the first phase and again before the second phase; these were the German version of the State Self-Esteem Scale (SSES; Heatherton and Polivy, 1991; Rudolph et al., in preparation) (α = 0.81) and the German Version of the Revised Competitive State Anxiety Inventory (Cox et al., 2003), the “Wettkampfangst-Inventar-State” (WAI-S; Ehrlenspiel et al., 2009) (α = 0.75). The WAI-S consists of 12 items on three separate dimensions: cognitive (α = 0.79), bodily anxiety (α = 0.75), and self-confidence (α = 0.86). After completing the ball-throwing task, participants filled out another questionnaire evaluating their task-related motivation (α = 0.54), confidence (α = 0.64), risk taking (α = 0.54), and degree of distraction (α = 0.83), each assessed with two items on five-point Likert scales. Participants assigned to one of the music conditions were also asked about their liking of and familiarity and identification with the music, assessed with single items on a five-point Likert scale.

## Results

Our analytic strategy was to approach each of the hypotheses individually before comparing potential interaction effects or relationships between them. Therefore, we conducted three independent analyses of variance (ANOVAs) for each of the hypothesized effects. Additional *post-hoc* analyses allowed us to further explore our findings and to formulate new hypotheses. Also, music ratings were investigated, which allowed us to compare both music conditions in terms of liking, familiarity, and identification. First, we investigated whether important covariates were equally distributed between the experimental conditions. No significant differences were observed with regard to age, gender, experience with ball-throwing activities (e.g., handball or volleyball), perceived ball-throwing efficacy, or risk propensity in sports and leisure, age: *F*_(2, 147)_ = 0.17, *p* = 0.85; gender: $\chi_{\left(2,\;\text{N}=149\right)}^{2}=2.02$, *p* = 0.36; previous ball-throwing activities: $\chi_{\left(2,\;\text{N}=150\right)}^{2}=0.59$, *p* = 0.74; perceived ball-throwing efficacy: *F*_(2, 143)_ = 0.19, *p* = 0.83; risk propensity *F*_(2, 147)_ = 0.39, *p* = 0.68. In addition, with regard to the task evaluation, no significant differences between experimental conditions were observed, motivation: *F*_(2, 146)_ = 1.16, *p* = 0.20; confidence: *F*_(2, 146)_ = 1.16, *p* = 0.20; risk taking: *F*_(2, 147)_ = 1.87, *p* = 0.16; distraction: *F*_(2, 147)_ = 2.45, *p* = 0.09.

### Self-evaluative cognition

To test whether motivational music enhanced self-evaluative cognitions, gain scores for each of the two self-report measures were calculated by subtracting pre-test scores from the scores obtained at the midpoint of the behavioral task (before the second phase of the study). An ANOVA revealed no significant differences in changes of state self-esteem between the control group (*M* = −2.46, *SD* = 6.56), experimenter-selected music (*M* = −1.06, *SD* = 5.85), and self-selected music (*M* = −1.59, *SD* = 6.60) conditions, *F*_(2, 140)_ = 0.63, *p* = 0.54, $\eta_{p}^{2}$ = 0.009. Also, no significant differences were found on any of the WAI-S scales across experimental conditions, cognitive anxiety: *F*_(2, 144)_ = 0.53, *p* = 0.59, $\eta_{p}^{2}$ = 0.007; bodily anxiety: *F*_(2, 146)_ = 1.18, *p* = 0.18, $\eta_{p}^{2}$ = 0.023; self-confidence: *F*_(2, 146)_ = 0.63, *p* = 0.53, $\eta_{p}^{2}$ = 0.009.

**Figure 2:**
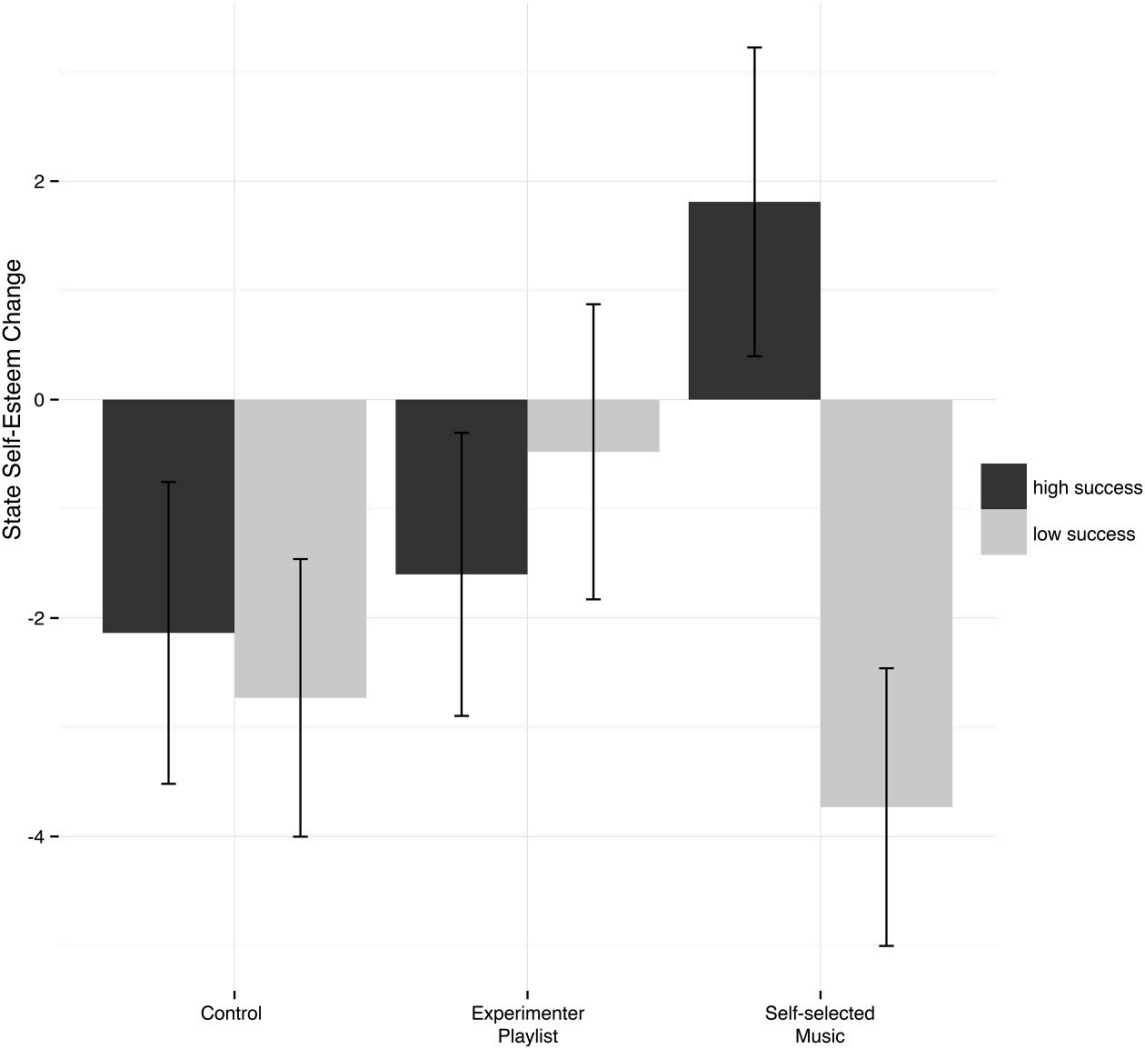
Estimated marginal means and standard error bars for state self-esteem gain scores by hitting success (high/low) across three conditions.

An additional *post-hoc* analysis was conducted to test whether success during the first phase of the behavioral task influenced self-evaluations. To this end, a new median split factor was computed based on the hitting scores obtained in the first phase of the task. A two-way ANOVA revealed a significant interaction of changes in self-esteem by ball-throwing success across experimental conditions (see Figure 2), *F*_(2, 137)_ = 3.347, *p* = 0.038, $\eta_{p}^{2}$ = 0.047. No significant main effects for condition [*F*_(2, 137)_ = 0.776, *p* = 0.46, $\eta_{p}^{2}$ = 0.011] or ball-throwing success [*F*_(2, 137)_ = 2.361, *p* = 0.13, $\eta_{p}^{2}$ = 0.017] were observed.

### Performance

A participant's success in ball-throwing performance was defined as the average performance across all 70 trials of the first phase of the task, measured as the sum of all successful trials. Thus, in order to analyze the effect of the experimental treatment on performance, sum scores for successful trials per participant for the first phase of the ball-throwing task were calculated. A one-way ANOVA did not indicate any significant differences in hitting scores between the control group (*M* = 23.82, *SD* = 6.34), the experimenter-selected music condition (*M* = 25.29, *SD* = 6.95), and the self-selected music condition (*M* = 24.65, *SD* = 5.17), *F*_(2, 147)_ = 0.70, *p* = 0.50, $\eta_{p}^{2}$ = 0.009. Although important covariates with regard to performance (e.g., ball-game experience, ball-throwing efficacy) were equally distributed between conditions, we conducted an additional analysis of covariance (ANCOVA) controlling for previous ball-throwing experience (yes/no) to reduce any type II error. The results revealed no increase in the effect of motivational music on performance [*F*_(2, 146)_ = 0.37, *p* = 0.69, $\eta_{p}^{2}$ = 0.005], but previous experience significantly predicted performance [*F*_(1, 146)_ = 5.30, *p* = 0.02, $\eta_{p}^{2}$ = 0.035].

### Risk taking

**Figure 3:**
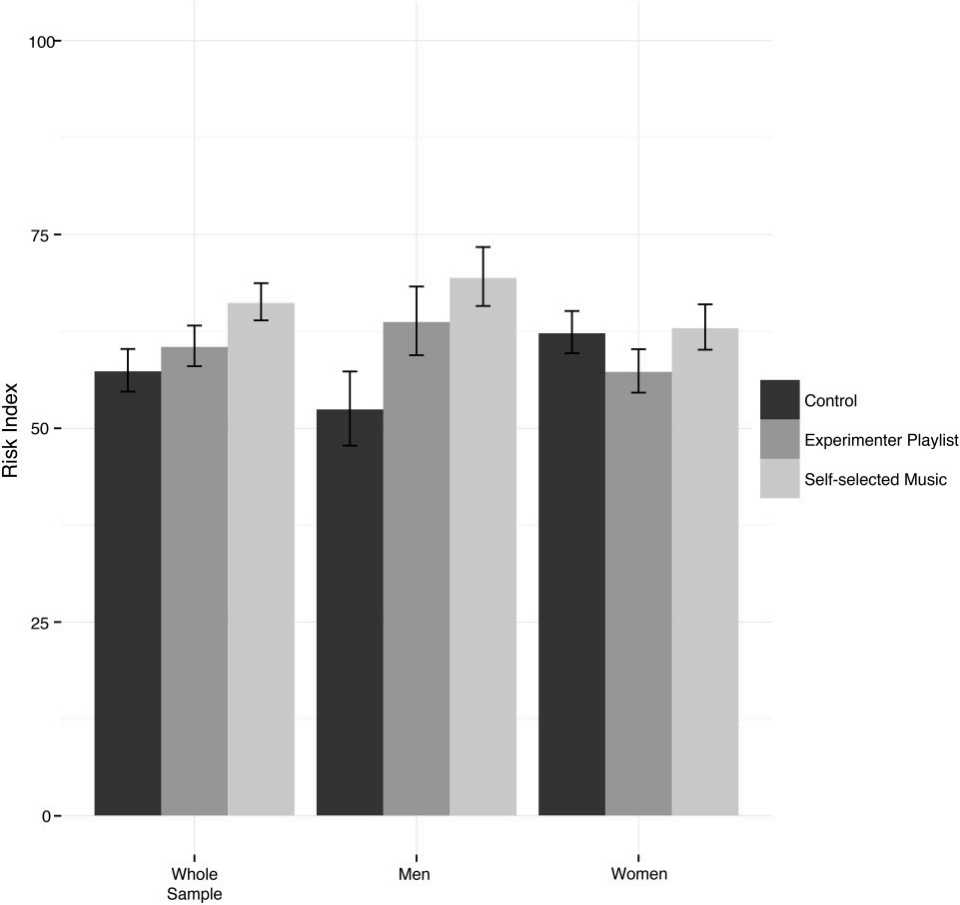
Estimated marginal means and standard error bars of success probability during the second phase of the ball-throwing task displayed for the whole sample and separately for male and female participants.

Risk taking was assessed using the mean hitting ratio from the first phase of the ball-throwing task corresponding to the distance participants chose during the second phase. Since the mean hitting ratio would become smaller when choices were riskier, a “risk index” was computed as the inversed score of the mean hitting ratio per participant, for practical reasons. A two-way ANOVA of the risk index by experimental condition and gender was conducted. The results indicated a significant main effect for condition, *F*_(2, 143)_ = 3.11, *p* = 0.048, $\eta_{p}^{2}$ = 0.042. There was no significant main effect for gender, *F*_(1, 143)_ = 0.12, *p* = 0.73, $\eta_{p}^{2}$ = 0.001. However, there was a significant interaction of risk-taking behavior and gender across conditions *F*_(2, 143)_ = 3.12, *p* = 0.047, $\eta_{p}^{2}$ = 0.042. Fischer's least significant difference method (LSD) was used for *post-hoc* comparisons of means between groups (see Figure 3). The results revealed no significant differences between the experimenter-selected music condition (*M* = 60.68, *SE* = 2.62), the no-music condition (*M* = 57.47, *SE* = 2.75) and the self-selected music condition (*M* = 66.32, *SE* = 2.40). The difference between the no-music condition and the self-selected music condition was marginally significant (*p* = 0.06).

Additional analyses of male and female subsets were conducted to resolve the interaction effect. Two independent ANOVAs indicated a significant simple effect of risk behavior across conditions for men but not for women, men: *F*_(2, 42)_ = 4.37, *p* = 0.02, $\eta_{p}^{2}$ = 0.319, women: *F*_(2, 101)_ = 1.15, *p* = 0.32, $\eta_{p}^{2}$ = 0.022. Using Fisher's LSD method, *post-hoc* comparisons of the estimated marginal means in the male subsample indicated that mean risk taking was significantly higher in the self-selected music condition (*M* = 69.58, *SE* = 3.59) than in the no-music condition (*M* = 52.54, *SE* = 4.52). However, there were no significant differences between the experimenter-selected music condition (*M* = 63.86, *SE* = 4.18) and either of the other two conditions (see Figure 3).

Another analysis was conducted to test how the level of risk affected overall success during the second phase of the task. A simple linear regression showed that a higher score on the risk index significantly predicted the total number of points earned, *R*^2^ = 0.07, *F*_(1, 148)_ = 12.03, *p* = 0.001. Thus, taking higher risks during the second phase predicted a higher gain in incentivized points and thus a higher reward for participants.

### Music ratings

For the music ratings we found significant differences between conditions, with consistently higher ratings for the self-selected music with regard to liking (*M*_Experi_. = 3.79, *SD* = 1.16; *M*_Self−selected_ = 4.67, *SD* = 0.71), familiarity (*M*_Experi_. = 4.35, *SD* = 0.81; *M*_Self−selected_ = 4.87, *SD* = 0.52), and identification (*M*_Experi_. = 3.25, *SD* = 1.04; *M*_Self−selected_ = 4.33, *SD* = 1.00), liking: *F*_(1, 98)_ = 21.27, *p* < 0.001, $\eta_{p}^{2}$ = 0.18; familiarity: *F*_(1, 98)_ = 14.19, *p* < 0.001, $\eta_{p}^{2}$ = 0.13; identification: *F*_(1, 98)_ = 27.69, *p* < 0.001, $\eta_{p}^{2}$ = 0.22. We also tested for a potential relationship between the music ratings and the two main outcome variables ball-throwing success and risk behavior. Bivariate Pearson correlations revealed no significant associations between music ratings and ball-throwing success (*r*_liking_ = 0.01, *N* = 100, *p* = 0.93; *r*_familiarity_ = −0.01, *N* = 100, *p* = 0.93; *r*_identification_ = −0.08, *N* = 100, *p* = 0.40) or risk behavior (*r*_liking_ = -0.14, *N* = 100, *p* = 0.17; *r*_familiarity_ = −0.15, *N* = 100, *p* = 0.13; *r*_identification_ = −0.05, *N* = 100, *p* = 0.60).

## Discussion

Use of music in sports and exercise has become a ubiquitous practice to enhance motivation, mood, and positive self-evaluations. While motivational music has proven to be ergogenic when applied during endurance tasks (Karageorghis and Priest, 2012a,b), the present study contributes to the existing literature by testing the effect of motivational music on (i) self-evaluative cognitions, (ii) performance, and (iii) risk-taking behavior. The ball-throwing paradigm allowed us to assess the effects of motivational music in a more naturalistic setting while retaining control over random allocation, music selection, and important covariates such as ball-throwing efficacy and trait risk taking.

Our main findings can be summarized as follows: First, music did not influence self-evaluative cognitions—neither trait self-esteem nor sport-related anxiety. However, music elevated state self-esteem among participants who were performing well in the ball-throwing task. Second, no performance-enhancing effect of motivational music for the ball-throwing task could be observed. But even though music did not improve performance, it also did not influence it negatively. Music could also have been distracting, preventing participants from focusing on the task. Yet music had no influence on overall performance, neither positive nor negative. Third, listening to motivational music enhanced risk-taking behavior, with self-selected playlists having a stronger effect than the experimenter-selected playlist. The effect was also more pronounced in men. Additionally, participants who made riskier choices earned higher monetary rewards.

### Self-evaluative cognitions

Although there is evidence in favor of a positive effect of music listening on self-esteem (Brown and Mankowski, 1993; Elvers et al., 2017), this study found neither a direct effect of motivational music on the enhancement of state self-esteem nor a reduction of cognitive and bodily anxiety. Since mean gain scores for state self-esteem were negative across conditions, the ball-throwing task itself seems to have had a negative impact on self-evaluative cognitions. It thus appears that the task had a stronger influence on self-evaluative cognitions and potentially overrode any positive effects of the music. Since the task appeared to be cognitively demanding, it might also have been the case that participants were not able to fully appreciate the music they were listening to, which might have further inhibited a positive effect of the music on the listeners.

Our finding that music had a positive impact on state self-esteem only when participants performed successfully during the first phase of the task highlights another interesting aspect of musical self-enhancement: It appears that music effectively amplified self-esteem only when congruent with a positive task performance and, in contrast, decreased state self-esteem among participants who were performing poorly. It might have been the case that listening to motivational music while performing the task poorly reminded participants that they could do better and emphasized the discrepancy between their expected and actual performance. This finding of a “congruency effect” with regard to state self-esteem aligns with other research that has identified congruency effects with regard to mood (Heimpel et al., 2002; Wood et al., 2009; Lee et al., 2013). It also converges with the theory of cognitive dissonance (Festinger, 1962), which posits that holding two or more incongruent cognitive beliefs leads to cognitive discomfort (i.e., mental stress). Cognitive dissonance has frequently been linked to self-evaluation processes and is assumed to influence momentary self-esteem (Steele and Liu, 1983; Tesser and Cornell, 1991). Listening to motivational music while performing poorly might have induced cognitive dissonance in participants, which led to lowered self-esteem.

### Performance enhancement

Although a considerable number of studies have investigated the effect of music in sports, there is little understanding of how music might exert ergogenic effects in sports performance. Here our aim was to extend previous findings by employing a study design that focusses on accuracy and motor coordination instead of endurance during a maximum-performance task (Karageorghis and Priest, 2012a,b). The ball-throwing task was more complex and involved both action planning and motor coordination. Here we did not find evidence for the hypothesis that motivational music would improve performance in a ball-throwing task, which was measured by the number of successful trials during the first phase of the ball-throwing task. We were able to confirm the robustness of this finding by ruling out potential confounds due to differences in ball-throwing efficacy or previous ball-throwing experience. Our findings do not align with qualitative accounts of the role of music in enhancing performance in ball sports (Bishop et al., 2007) or with studies with small sample sizes that suggest a performance-enhancing effect in ball sports (Silliman and French, 1993; Pates et al., 2003). Rather, they suggest that the performance-enhancing effect of music that has been documented in other domains appears not to be generalizable to tasks that are more focused on accuracy and motor coordination than on endurance. In comparison to maximum-performance tasks, the ball-throwing task involved more cognitive processing, mental focus, and attention, whereas running or cycling involve relatively little concentration. It therefore appears that a performance-enhancing effect of music is more likely to take place when performance of the task is relatively autonomous and does not require a lot of mental effort (Terry and Karageorghis, 2011). This would suggest that the induction of flow states may account for performance-enhancing effects of music (Silliman and French, 1993) but may have been inhibited in this study due to the relative complexity of the ball-throwing task.

### Risk-taking behavior

Several studies provide evidence that certain types of music evoke cognitive and affective changes in the listener that are likely to result in greater risk-taking behavior. Enhanced risk-taking induced by music has been observed in driving simulations (Brodsky, 2002; Dey et al., 2006) and also when a behavioral economics bidding paradigm was employed (Halko and Kaustia, 2015; Halko et al., 2015). Our findings converge with these studies and support the claim that listening to music promotes making risky decisions. Our study found the risk-promoting effect in response to motivational music that has specific characteristics (in the experimenter-selected music condition, e.g., motivational lyrics, fast tempi, high level of energy, positive emotional expression, etc.; see further Karageorghis et al., 1999, 2006) or that was selected for the specific purpose of motivation (self-selected music condition). We found that, compared to the no-music control condition, the self-selected music condition was most effective in promoting risky decision making, and this effect was more pronounced among male participants. Furthermore, a regression analysis showed that the overall earnings of incentivized points were positively predicted by the level of risk taking. This suggests that taking higher risks may be beneficial, since in our study taking higher risks led to higher monetary rewards. Being motivated to reach the threshold of one's own competency when making risk-related decisions may be especially important in the domain of sports. Based on our findings, we conclude that music presumably has an advantageous function in sports by promoting greater risk taking without exceeding the range of an athlete's abilities.

The risk-enhancing effect of music presumably relies on a different perception of risk-related decisions. Based on prospect theory (Kahneman and Tversky, 1979), this can be explained as follows: Listening to music may lead to a perception of choices involving risks in terms of potential gains rather than potential losses. This effect may be explained via some form of empathy (Zentner et al., 2008; Singer and Lamm, 2009; Clarke et al., 2015) or identification (Cohen, 2001). In a broader sense, motivational music can be characterized as expressing a high sense of self-confidence and risk-averse attitudes (e.g., see songs in Table 1), and when listening to such music, the listener empathizes and identifies with the singer and to a certain extent adopts these attitudes her- or himself (Elvers, 2016). A similar relationship has been documented in the field of media studies, where a positive association between the consumption of risk-promoting media content and risky driving behavior was found across different types of studies (correlational, experimental, longitudinal (Beullens et al., 2016). Mimetic and empathetic responses to music and other media content may, however, not always occur and may have different degrees of intensity. Preference judgements have been identified as important predictors of empathic responses (Egermann and McAdams, 2013), suggesting that the reward value of the music plays an important role in determining whether and to what extent the expressed attitude and behavior will be adopted by the listener.

According to Lang (2006) Limited Capacity Model of Motivated Mediated Message Processing (LC4MP), “some messages can be considered to be more motivationally relevant, namely, those that are implicitly or explicitly associated with pleasure or danger” (Beullens et al., 2016, p. 185). Thus, behavioral approach/inhibition tendencies that are responsive to music-derived pleasure may count as potential mediators of the effect of motivational music on risk behavior. Neuroscientific studies have documented the relationship between music-induced pleasure and reward (Blood and Zatorre, 2001; Salimpoor et al., 2013, 2015), and recent correlational evidence has shown that high reward sensitivity is linked to higher involvement with and stronger positive responses to music (Loxton et al., 2016). Thus, in our study, listening to music may have evoked empathic responses to the overt positive self-portrayals conveyed by the music, which in turn may have promoted riskier decision making, with this relationship moderated by how pleasurable the music was experienced as being. This interpretation is supported by our finding that in the self-selected music condition, both higher liking ratings and higher degrees of risk taking were observed than in the experimenter-selected playlist condition.

The gender differences in risk-taking behavior that we found in our study align with what previous studies have documented. In a meta-analysis, Byrnes et al. (1999) showed in that risk taking across domains, men take higher risks than women. These differences are even more pronounced when the risk taking involves physical skills. Research from behavioral economics has identified gender differences with regard to the willingness to compete. Niederle and Vesterlund (2007) found that men select the option of competing in a tournament when they have the opportunity to do so twice as much as women do. Niederle and Vesterlund (2007) explained these gender differences in terms of men being more overconfident than women. Since we found no main effect of gender on risk taking, it is likely that differences in confidence evaluations were responsible for the effects identified in our study.

## Conclusion

Music is a ubiquitous and pervasive cultural artifact that serves as a resource for pleasurable experiences (Zatorre and Salimpoor, 2013) and allows us to fulfill various types of psychosocial needs (DeNora, 1999, 2000). It interferes with our affective and cognitive attitudes and dispositions and eventually influences how we behave and interact with one another. This *power of music* was already recognized in antiquity (Woerther, 2008) and has stimulated scholars ever since to explore and investigate this phenomenon. We aimed to contribute to an understanding of this *power* by investigating a contemporary practice of music listening wherein people use music to stimulate their cognitions and behaviors. The use of motivational music in sports and exercise to self-enhance, improve performance, and increase risky behavior is well-documented by ample anecdotal evidence as well as correlational and qualitative accounts. However, our findings reflect the intricateness of these phenomena, as only one of our three hypotheses was confirmed. Although we gathered evidence in favor of a risk-enhancing effect of music, more research is needed in order to improve the robustness of this finding. Another issue is the administration of motivational music in experimental settings. In this study, it was decided to present the music during the task. However, another option would be to only present motivational music before the task that is examined. Furthermore, research is also needed to address the potential mechanisms that may account for the finding; we believe that music's ability to induce pleasure as well as its function with respect to self-enhancement serve as promising candidates for future investigations.

## Author contributions

PE and JS both contributet significantly to the design of the study the data collection and analysis. PE wrote most of the manuscript but JS critically revised important parts of the manuscript.

### Conflict of interest statement

The authors declare that the research was conducted in the absence of any commercial or financial relationships that could be construed as a potential conflict of interest.

## Footnotes

^1^Three musicologists, a sound engineer, and an expert on popular music.
