# Supplementary material for: The Sound of Success: Investigating Cognitive and Behavioral Effects of Motivational Music in Sports
Source: Front Psychol. 2017 Nov 21;8:2026. doi: 10.3389/fpsyg.2017.02026 (PMC5702473; doi:10.3389/fpsyg.2017.02026)
Supplement: Supplementary file 1 [file Table1.DOCX]

Supplementary Material

Motivational music leads to higher risk-taking behavior

without improving ball game performance

Paul Elvers^1*^, Jochen Steffens^2*^

^1^Max Planck Institute for Empirical Aesthetics, Frankfurt am Main, Germany

^2^Audio Communication Group, Technische Universität Berlin, Berlin, Germany

*** Correspondence:**Paul Elvers, Grüneburgweg 14, 60322 Frankfurt am Main, Germany.
Email: paul.elvers@ae.mpg.de

# Supplementary Data: Description of the task to compile a playlist with motivational music in German and English

German version: „Bitte stellen Sie im Folgenden eine Playlist zusammen mit Ihren ganz persönlichen Lieblingsstücken, die auf Sie besonders motivierend wirken. Dies können Stücke sein, die Sie gerne beim Sport, oder beim Auto- oder Fahrradfahren hören. Egal, um welche Musik es sich handelt und wo Sie diese Musik hören, wichtig ist, dass diese Musik für Sie persönlich besonders motivierend wirkt. Motivierende Musik kann so erlebt werden, dass sie Ihnen Kraft verleiht, zu mehr Selbstbewusstsein führt oder Ihnen hilft, mit schwierigen Situationen besser umzugehen. Versuchen Sie also bitte hier Ihre ganz persönlichen Lieblingsstücke anzugeben, die auf Sie am stärksten motivierend wirken. Bitte geben Sie insgesamt 12 Titel an.“

English translation: “In the following section, please compile a playlist of your favorite musical pieces that you perceive as motivating. These could be pieces you listen to while doing sports, driving a car, or riding a bike. No matter what kind of music it is or where you like to listen to this music, the only thing that is important is that you perceive these musical pieces as motivating. Musical pieces can be perceived as inducing feelings of power, enhance self-confidence, or that allow you to master challenging situations. Please try to compile your personally favorite songs that you perceive as motivating. Please list 12 musical pieces.”
